# The Expression of BAFF, APRIL and TWEAK Is Altered in Eczema Skin but Not in the Circulation of Atopic and Seborrheic Eczema Patients

**DOI:** 10.1371/journal.pone.0022202

**Published:** 2011-07-13

**Authors:** Yunying Chen, Sara Lind Enoksson, Catharina Johansson, Maria A. Karlsson, Lena Lundeberg, Gunnar Nilsson, Annika Scheynius, Mikael C. I. Karlsson

**Affiliations:** 1 Clinical Allergy Research Unit, Department of Medicine Solna, Karolinska Institutet and Karolinska University Hospital Solna, Stockholm, Sweden; 2 Dermatology Unit, Department of Medicine Solna, Karolinska Institutet and Karolinska University Hospital Solna, Stockholm, Sweden; 3 Clinical Immunology and Allergy Unit, Department of Medicine Solna, Karolinska Institutet and Karolinska University Hospital Solna, Stockholm, Sweden; University of Brescia, Italy

## Abstract

The TNF family cytokines BAFF (B-cell activating factor of the TNF family) and APRIL (a proliferation-inducing ligand) are crucial survival factors for B-cell development and activation. B-cell directed treatments have been shown to improve atopic eczema (AE), suggesting the involvement of these cytokines in the pathogenesis of AE. We therefore analyzed the expression of these TNF cytokines in AE, seborrheic eczema (SE) and healthy controls (HC). The serum/plasma concentration of BAFF, APRIL and a close TNF member TWEAK (TNF-like weak inducer of apoptosis) was measured by ELISA. The expression of these cytokines and their receptors in skin was analyzed by quantitative RT-PCR and immunofluorescence. Unlike other inflammatory diseases including autoimmune diseases and asthma, the circulating levels of BAFF, APRIL and TWEAK were not elevated in AE or SE patients compared with HCs and did not correlate with the disease severity or systemic IgE levels in AE patients. Interestingly, we found that the expression of these cytokines and their receptors was altered in positive atopy patch test reactions in AE patients (APT-AE) and in lesional skin of AE and SE patients. The expression of APRIL was decreased and the expression of BAFF was increased in eczema skin of AE and SE, which could contribute to a reduced negative regulatory input on B-cells. This was found to be more pronounced in APT-AE, the initiating acute stage of AE, which may result in dysregulation of over-activated B-cells. Furthermore, the expression levels of TWEAK and its receptor positively correlated to each other in SE lesions, but inversely correlated in AE lesions. These results shed light on potential pathogenic roles of these TNF factors in AE and SE, and pinpoint a potential of tailored treatments towards these factors in AE and SE.

## Introduction

Atopic eczema (AE) is a chronic inflammatory skin disorder and known to be caused by a combination of multiple factors, including genetic predisposition, skin barrier dysfunction, immunological deviation and environmental allergens [1]. Most AE patients have elevated concentration of total and allergen-specific serum IgE [2]. Some patients also have IgE against self-antigens, such as manganese superoxide dismutase (MnSOD) [2], [3]. The lipophilic yeast *Malassezia* belongs to the commensal skin microflora, however, it can induce specific IgE and T-cell reactivity in AE patients [2], [3]. Seborrheic eczema (SE) is another chronic inflammatory skin disorder associated with *Malassezia* reactivity but without IgE production [4].

Although the exact role of B-cells in AE is not fully understood, the contribution of B-cells to AE etiopathogenesis has become evident by studies showing that B-cell-depleting treatment with anti-CD20 Ab improved AE skin lesions with reduced mRNA expression of IL-5 and IL-13 and decreased infiltration of T and B-cells in skin, whereas total and allergen-specific IgE levels were not reduced, suggesting other functions than Ab production of B-cells in the disease mechanisms [5]. It has recently become appreciated that aberrant regulation and activation of B-cells result in chronic inflammatory and autoimmune-mediated disorders. They contribute to the disease pathogenesis not only by being the Ab producers, but also as APCs (antigen-presenting cells) and cytokine/chemokine producers [6], [7]. Accordingly, B-cell directed therapy, including Abs against B-cell specific markers and inhibitors of survival and signalling factors for B-cells, is currently introduced for the treatment of inflammatory and autoimmune diseases [6], [7].

The TNF ligand members BAFF (B-cell activating factor of the TNF family) and APRIL (a proliferation-inducing ligand) are two crucial survival factors for peripheral B-cells [8]. They can promote Ab class switching independently of the CD40/CD40L pathway [8]. BAFF and APRIL are expressed mainly by innate immune cells, to a less extent by T-cells and activated B-cells, as well as non-haematopoietic tissue resident cells [8]. BAFF and APRIL share the receptors TACI (transmembrane activator and calcium-modulator and cyclophilin ligand interactor) and BCMA (B-cell maturation antigen). In addition, BAFF binds to BAFFR and APRIL interacts with heparan sulphate proteoglycans [8]. BAFF is expressed as both surface-bound and soluble factors, whereas APRIL is processed inside the cell and released as a soluble protein [8]. However, APRIL can be attached to the cell surface by being a natural fusion protein with TWEAK (TNF-like weak inducer of apoptosis), called TWE-PRIL, sharing receptors with APRIL [9]. Thus, these factors form a network of mediators interacting with an overlapping set of receptors.

In humans, increased levels of BAFF and/or APRIL in serum or target tissues have been described in a number of autoimmune conditions and often correlated to disease progression [8]. In allergic diseases, so far, an elevated serum level of BAFF has been suggested as a diagnostic parameter for asthma [10]. In AE, an increased serum BAFF level in children has been reported [11]. However, another group has published data in adult AE patients having an elevated serum level of APRIL, but not BAFF [12].

When not fused to APRIL, full length TWEAK is a multifunctional cytokine, regulating cell proliferation, migration, differentiation, apoptosis, angiogenesis and inflammation [13], playing either beneficial or detrimental biological effects in mouse models, depending on the tissue injury or disease model that is used [14]. Its receptor Fn14 (Fibroblast growth factor-inducible 14) is expressed by many cell types, but absent on lymphocytes [13]. The expression of TWEAK and Fn14 is relatively low in normal tissues and up-regulated by tissue injury or disease [13]. It has recently been reported that TWEAK and TNF-α can cooperate in the induction of keratinocyte apoptosis, suggesting a role of TWEAK in eczema formation of AE [15] . Apart from this, TWEAK/Fn14 interaction in human keratinocytes has also been shown to induce the production of the chemokine RANTES, suggesting the potential involvement of TWEAK/Fn14 in the pathophysiology of inflammatory skin disorders [16].

To define the role of BAFF, APRIL and TWEAK in inflammatory skin disease, we characterized their expression in the circulation and the skin in AE, SE and HC. To get a more complete picture of the dynamic regulation of these factors, positive atopy patch test reactions in AE patients (APT-AE) was included, which mimics the early stage of AE lesions [17], [18]. Our results demonstrate that unlike other inflammatory diseases, the levels of BAFF, APRIL and TWEAK are not elevated in the circulation and do not correlate with the severity of AE. Instead, the levels and expression patterns of these cytokines are changed in eczema skin of both AE and SE compared to skin of HC, showing an altered balance in the eczema skin for these TNF members.

## Results

### Circulating levels of BAFF, APRIL and TWEAK are not elevated in AE or SE patients

**Figure 1:**
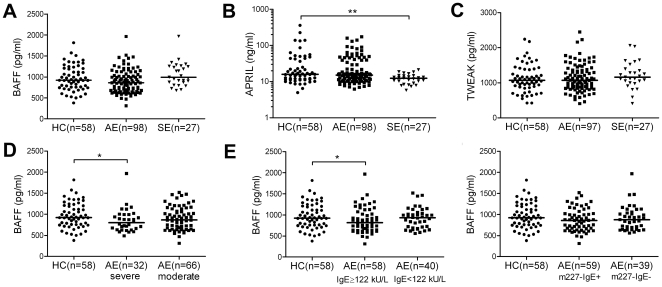
Plasma/serum levels of BAFF, APRIL and TWEAK in HC and patients with AE or SE. (A) BAFF, (B) APRIL and (C) TWEAK measured by ELISA in HC and patients with AE or SE. (D–F) Plasma levels of BAFF in subgroups of AE patients with (D) severe or moderate AE, (E) with/without increased total plasma IgE and (F) with/without *Malassezia* (m227)-specific plasma IgE. Data shown as individual patients or healthy controls, horizontal bar indicates the median, *p<0.05, **p<0.01 by Mann-Whitney test.

First, we measured plasma/serum levels of BAFF, APRIL and TWEAK by ELISA. We found that these cytokines were not elevated in AE or SE patients compared to HC (Fig. 1A–C). When investigating subgroups of AE patients (Fig. 1D–F), the plasma levels of BAFF were found to be decreased in patients with severe AE (Fig. 1D), as well as in the subgroup with elevated total IgE (Fig. 1E). However, the plasma/serum levels of these cytokines did not correlate with the SCORAD index or with the plasma levels of total IgE or *Malassezia-*specific IgE in AE patients (Table 1). Since the circulating levels of these factors have been found to be increased in autoimmune disease [8], [13], we analyzed a subgroup of AE patients to elucidate whether their specific IgE levels to human MnSOD (n = 5) [19] correlated with the circulating levels of these cytokines, however, no correlation was found (data not shown).

**Table 1:** Correlations between the serum/plasma levels of BAFF, APRIL, TWEAK and SCORAD, plasma levels of total-IgE or *Malassezia*-IgE in patients with AE, respectively.

Correlation	R value	P value
BAFF and SCORAD	−0.114	0.263
BAFF and total IgE	−0.179	0.078
BAFF and *Malassezia*-IgE	−0.120	0.240
APRIL and SCORAD	0.063	0.539
APRIL and total IgE	0.130	0.202
APRIL and *Malassezia*-IgE	0.094	0.360
TWEAK and SCORAD	0.018	0.861
TWEAK and total IgE	−0.078	0.449
TWEAK and *Malassezia*-IgE	−0.099	0.337

Correlations were assessed with the Spearman's rank correlation (n = 97–98).

### The mRNA expression of BAFF is increased in SE and APT-AE, but not in AE lesions

To analyze the expression of BAFF, APRIL and TWEAK in eczema lesions, skin biopsies from AE and SE patients as well as HC were randomly selected from the respective groups in table 2 (Table S1). The samples were analyzed by quantitative RT-PCR and verified for protein expression by immunofluorescence.

**Table 2:** Characterization of patients with AE or SE and healthy controls.

	Healthy controls	AE patients	SE patients
Total number of participants	58	99	27
Gender (female/male)	36/22	51/48	5/22
Age (years)	37 (18–64)^a^	28 (18–65)^a^	39 (22–63)^a^
SCORAD	-	35 (15–69)^a^	-
Mild AE/moderate AE/severe AE^b^	-	(0/66/33)	-
Plasma total IgE (kU/L)	20 (3.2–140)^a^	280 (2–15100)^a^	17 (2–390)^a^
Plasma *Malassezia*-IgE	0%	60%	0%
Phadiatop® positive	0%	80%	22%
Asthma and/or rhinitis	0%	74%	22%

a ) Median (range);

b ) Defined according to the objective SCORAD index (range 0–83; mild <15, moderate = 15–40, severe >40) [37].

**Figure 2:**
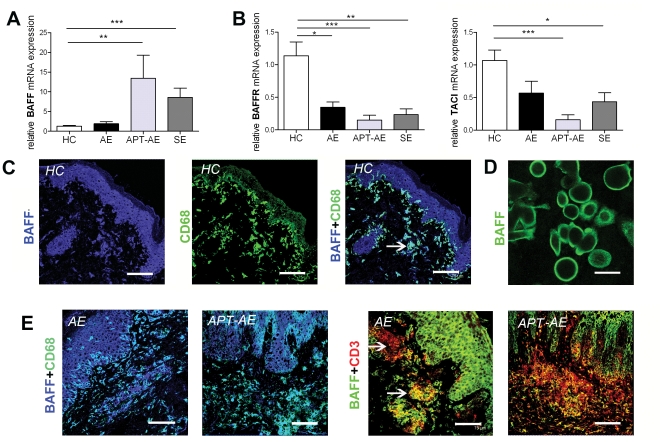
Expression of BAFF in skin of HC and lesional skin of AE and SE determined by quantitative RT-PCR and immunofluorescence. (A–B) Relative mRNA expression of BAFF, BAFFR and TACI in skin of HC (n = 9), lesional skin of AE (n = 9), APT-AE (n = 6) and SE (n = 6). Data shown as mean ± SEM, *p<0.05, **p<0.01, ***p<0.001 by Mann-Whitney test. (C) Representative picture of double-immunostaining with anti-BAFF and anti-CD68 mAb on sections of skin biopsies from HC (bar = 75 µm). The arrow indicates representative cells double positive for anti-BAFF and anti-CD68 staining. (D) Immunostaining with anti-BAFF mAb on cultured human keratinocytes (bar = 30 µm). (E) Representative pictures of double-immunostaining with anti-BAFF and anti-CD68/anti-CD3 mAb on sections of lesional AE skin and APT-AE skin specimens (bar = 75 µm). The upper arrow indicates the red cells stained with only anti-CD3 mAb. The lower arrow indicates the yellow cells stained with both anti-CD3 and anti-BAFF mAb.

We found that mRNA expression of BAFF was about 10 times higher in APT-AE and about 7 times higher in SE lesions compared to HC (Fig. 2A). The mRNA levels of BAFFR and TACI were decreased in all the inflammatory conditions (Fig. 2B). Since these two receptors are known to be mainly expressed by B-cells [8], we normalized the mRNA levels of BAFFR and TACI to the mRNA levels of a B-cell specific marker CD19, the relative mRNA expression of these two receptors appeared similar in all groups (data not shown). We next verified the protein expression pattern of BAFF in skin. Immunostaining indicated that the cellular origins of BAFF in healthy skin were keratinocytes and cells in dermis co-stained with anti-CD68 mAb (Fig. 2C). The expression of BAFF by keratinocytes in healthy skin was confirmed by immunostaining of human keratinocytes (Fig. 2D). However, immunostaining for BAFFR and TACI expression on skin sections and on cultured keratinocytes revealed that these two receptors were not expressed by keratinocytes (data not shown). In lesional skin of AE, APT-AE and SE, besides the keratinocytes, subpopulations of the infiltrating CD3^+^ T cells and CD68^+^ macrophages also expressed BAFF, as shown by double-immunostaining on skin sections (Fig. 2E).

### The mRNA expression of APRIL is decreased in lesional skin of AE, APT-AE and SE

**Figure 3:**
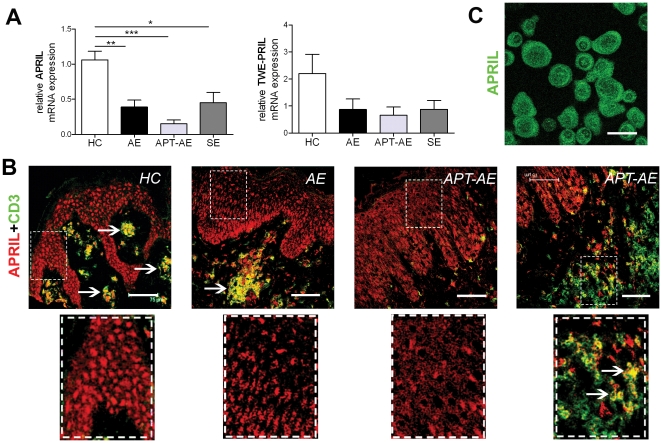
Expression of APRIL in skin of HC and lesional skin of AE and SE determined by quantitative RT-PCR and immunofluorescence. (A) Relative mRNA expression of APRIL and TWE-PRIL in skin of HC (n = 9), lesional skin of AE (n = 9), APT-AE (n = 6) and SE (n = 6). Data shown as mean ± SEM, *p<0.05, **p<0.01 by Mann-Whitney test. (B) Representative pictures of double-immunostaining with anti-APRIL and anti-CD3 mAb on sections of skin biopsies from HC, lesional AE skin and APT-AE skin specimens (bar = 75 µm). The arrows indicate representative yellow cells double positive for anti-CD3 and anti-APRIL staining. (C) Cultured human keratinocytes immunostained with anti-APRIL mAb (bar = 30 µm).

In contrast to BAFF, the relative mRNA levels of APRIL were decreased in lesional skin of AE, APT-AE and SE, compared to HC (Fig. 3A). Similarly, “TWE-PRIL”, the cell surface bound form of APRIL, showed a tendency of lower expression in lesional skin (Fig. 3A). In healthy skin, APRIL was expressed by keratinocytes and by cells in dermis co-stained with anti-CD3 (Fig. 3B) or anti-CD68 mAb (data not shown). We confirmed the expression of APRIL in healthy keratinocytes by immunostaining of cultured human keratinocytes, which showed a similar intracellular expression pattern as found in HC skin (Fig. 3C) and was different from the cell membrane associated staining pattern of BAFF seen in figure 2. The expression of APRIL by keratinocytes in lesional skin of AE and APT-AE was decreased and among the infiltrating CD3^+^ T-cells in APT-AE, only a small subpopulation was found to express APRIL (Fig. 3B), which was in line with the decreased mRNA expression of APRIL observed in these inflammatory skin lesions.

### The mRNA expression of Fn14 is increased in AE and APT-AE skin lesions

**Figure 4:**
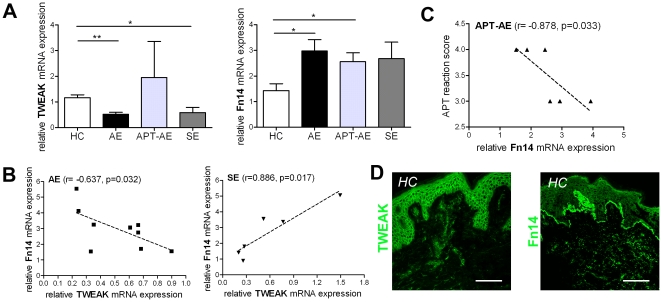
Expression of TWEAK in skin of HC and lesional skin of AE and SE determined by quantitative RT-PCR and immunofluorescence. (A) Relative mRNA expression of TWEAK and Fn14 in skin of HC (n = 9), lesional skin of AE (n = 9), APT-AE (n = 6) and SE (n = 6). Data shown as mean ± SEM, *p<0.05, **p<0.01 by Mann-Whitney test. (B) Correlation of relative mRNA levels of TWEAK and Fn14 in AE (n = 9) and SE (n = 6) skin specimens. (C) Correlation of relative mRNA levels of Fn14 and APT reaction scores in APT-AE (n = 6) skin specimens. The correlations were analyzed using Spearman's rank correlation. (D) Representative picture of immunostaining with anti-TWEAK or anti-Fn14 mAb on sections of skin specimens from HC (bar = 75 µm).

Local expression of TWEAK and its receptor Fn14 have been shown to be increased in target tissues of several diseases [13]. Here, we found a decreased mRNA expression of TWEAK in lesional skin of AE, APT-AE and SE (Fig. 4A), whereas the mRNA expression of Fn14 showed having an increased tendency in lesional skin of AE, SE and APT-AE (Fig. 4A). Interestingly, the mRNA levels of Fn14 and TWEAK positively correlated to each other in SE, while these factors correlated inversely in AE (Fig. 4B). Furthermore, the mRNA levels of Fn14 in APT-AE correlated inversely with the APT scores (Fig. 4C). The protein expression pattern of TWEAK in skin specimens from all groups was similar, where TWEAK was expressed by keratinocytes and cells in dermis (Fig. 4D). The expression of Fn14 was in the dermal-epidermal basement membrane zone in both healthy skin of HC (Fig. 4D) and skin of AE, SE and APT-AE. Primary keratinocytes in culture did not stain with anti-TWEAK mAb and anti-Fn14 mAb (data not shown), which suggest that the local environment is important for their expression.

### The mRNA expression of BAFF in cultured keratinocytes is regulated by IFN-γ and IL-27 and is correlated with that of IL-18 in lesional AE skin

**Figure 5:**
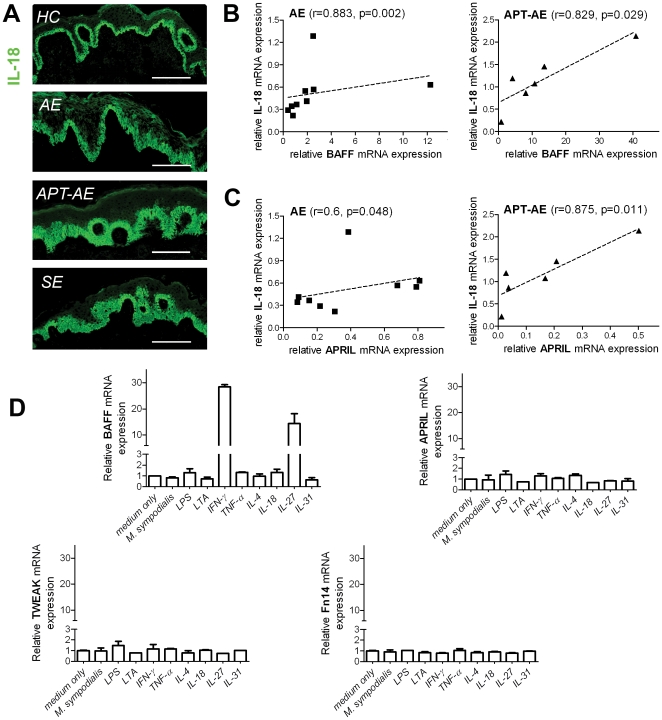
Expression of IL-18 in AE, SE and HC skin determined by immunofluorescence and the regulation of mRNA expression of BAFF, APRIL, TWEAK and Fn14 in healthy keratinocytes by IL-18 and other eczema related stimuli. (A) Representative pictures of immunostaining with anti-IL-18 mAb on sections of skin biopsies from HC, lesional skin of AE, APT-AE and SE (bar = 150 µm). (B) Correlation of relative mRNA levels of BAFF and IL-18 in AE (n = 9) and APT-AE (n = 6) skin specimens. (C) Correlation of relative mRNA levels of APRIL and IL-18 in AE (n = 9) and APT-AE (n = 6) skin specimens. The correlations were analyzed using Spearman's rank correlation. (D) Relative mRNA expression of BAFF, APRIL, TWEAK and Fn14 in healthy keratinocytes cultured for 24 h with indicated stimuli. Data are shown as mean ± SEM (n = 3) from two separate experiments.

The correlation between the protein levels of BAFF, APRIL and TWEAK in the circulation and mRNA levels in the skin was analyzed, however, no correlation was found (data not shown), suggesting that the systemic expression of these cytokines is not connected to the levels in the local environment in eczema skin. We and others have previously shown that the plasma level of IL-18, a product of activation of the inflammasome, is increased in AE patients [20], [21], [22]. In addition, the expression of IL-18 has been found to be increased in inflamed skin [23]. Thus, as a measure of local inflammation for means of correlation, we investigated the expression of IL-18 in lesional skin of AE and SE. IL-18 was found to be expressed at the level of the single basal layer of keratinocytes in HC, whereas expression in the suprabasal layers of keratinocytes was observed in lesional skin of AE, and particularly in APT-AE and SE (Fig. 5A). The mRNA level of IL-18 was found to correlate with BAFF and APRIL in lesional skin of AE and APT-AE (Fig. 5B, C), but not in HC and SE lesions (data not shown). No correlation between IL-18 and TWEAK was found (data not shown). Since we found expression of BAFF and APRIL in healthy keratinocytes (Fig. 2D and Fig. 3C), we wanted to investigate if this expression could be regulated by IL-18, as well as other stimuli involved in the pathogenesis of AE. We found that IFN-γ and IL-27 dramatically increased the mRNA level of BAFF but not that of APRIL, TWEAK and Fn14 in keratinocytes stimulated for 24 hours (Fig. 5D). The other stimuli tested, *M. sympodialis* extract, LPS, LTA (Lipoteichoic acid), TNF-α, IL-4, IL-18 and IL-31, did not influence the mRNA levels analyzed (Fig. 5D).

## Discussion

The role of B-cells in the pathology of allergy is undisputable, and their contribution beyond IgE production has recently started to be appreciated [6]. However, the factors influencing B-cell activation and homeostasis in allergic diseases, in both systemic and target tissue environments, need to be characterized. Here, we sought to define the role for BAFF, APRIL and TWEAK in inflammatory skin disease by comparing the systemic levels and skin expression in AE and SE patients as well as HCs.

Unlike other inflammatory diseases including autoimmune diseases and asthma [8], [10], we found that the circulating levels of BAFF and APRIL were not elevated in patients with AE and they did not correlate with AE severity and plasma levels of total IgE or *Malassezia*-specific IgE. Notably, a recent publication has shown that the serum levels of BAFF were increased in children with AE [11], but this was not the case in adult patients [12], in agreement with our results. These findings suggest that BAFF might play a role at an early stage of AE but not at a later adult stage. Indeed, this seems to be the case in the local skin environment, where the mRNA expression of BAFF is found to be dramatically increased in APT-AE, which mimics the acute lesion.

Matsushita *et al* reported an increased level of APRIL in AE patients compared to HCs [12], which is not found in the cohort of patients investigated here. The reason for this is unclear, but might suggest that larger cohorts and multiple studies are needed to settle if levels of these factors are really altered in AE patients. On the other hand, our results show that AE and SE, two different chronic inflammatory skin diseases, can be distinguished by the systemic APRIL levels. Patients with SE displayed lower systemic APRIL concentration compared to HCs. The pathological role of the decreased serum APRIL level in SE remains to be determined.

The keratinocytes in skin, besides the barrier function, can also secrete numerous cytokines, chemokines and some of the growth factors in an autocrine manner regulating barrier homeostasis, and this ability of keratinocytes has been shown to be dysregulated in AE lesional skin [24]. It is known that BAFF and APRIL can be expressed by resident cells in several kinds of tissues [8]. We report here that healthy keratinocytes can also synthesize and express BAFF and APRIL, both *in vivo* and *ex vivo*, and in lesional skin of AE, APT-AE and SE the keratinocytes have impaired APRIL expression compared to HCs. Different from other TNF ligands, APRIL only functions as a soluble protein. By immunohistological analysis, we confirmed the intracellular expression pattern of APRIL in keratinocytes both *in vivo* and *ex vivo*, which is different from the cell membrane associated expression pattern of BAFF. We then studied the regulation of BAFF and APRIL *in vitro* in keratinocytes using some factors involved in the pathogenesis of eczema and we found that the BAFF mRNA level was dramatically increased by IFN-γ and IL-27. IL-27 is a member of the IL-12 family, produced by activated APCs [25]. The increased expression of IFN-γ and IL-27 in chronic eczema skin lesions has been demonstrated before [1], [25], and both IFN-γ and IL-27 have been shown to greatly induce the production of CXCL 9–11 in normal human keratinocytes [26], [27], the chemokines known to attract Th1 cells, triggering and sustaining skin inflammation [26], [27]. Here we suggest that IFN-γ and IL-27 can amplify the skin inflammation not only through the regulation of the chemokine production in keratinocytes, but also through the regulation of BAFF expression in these cells.

By immunostaining we did not found the expression of TACI or BAFFR by keratinocytes both *in vivo* and *ex vivo*, indicating that the expression of BAFF and APRIL by keratinocytes is not regulated by feedback autocrine mechanism. BAFF and APRIL thus presumably have no regulatory role for the homeostasis of keratinocytes. TACI and BAFFR are mainly expressed by B-cells [8] and the presence of B-cells in normal skin and the infiltration of B-cells in AE eczema skin have been demonstrated by Simon *et al* [5]. We could detect the expression of these two receptors in skin, however, the mRNA level of these two receptors in inflamed skin of APT-AE, AE and SE was not found to be increased. The reason for this could be the dilute effect by the influx of a large number of T-cells and macrophages in inflammatory skin lesions. Indeed, when the mRNA levels of these two receptors were normalized to the expression level of a B-cells maker CD19, the relative mRNA expression of TACI and BAFFR appeared similar in all groups.

APRIL is a close homologue to BAFF and they share many functions and receptors. Here we demonstrate that opposite to BAFF, the expression of APRIL, as well as the cell surface APRIL hybrid TWE-PRIL, is down-regulated in the skin lesions of APT-AE, AE and SE. It has been shown that APRIL binding to TACI delivers a negative signal to B-cells [28]. Since APRIL does not bind to BAFFR, it does not deliver a BAFFR mediated positive signal to B-cells. Therefore, the decreased expression of APRIL and TWE-PRIL together with the increased BAFF expression in eczema skin could contribute to a reduced negative regulatory input on B-cells, which is more pronounced in the initiating acute stage of AE, resulting in dysregulation of over-activated B-cells in lesional skin of AE and SE. Our data of the down-regulation of APRIL and the up-regulation of BAFF in the eczema skin of AE and SE is also important for guiding potential clinical treatment. When targeting BAFF and APRIL in the therapy for AE and SE, the agents neutralizing only BAFF but having no effect on APRIL might be more suitable than those targeting both BAFF and APRIL.

The up-regulation of IL-18 in suprabasal keratinocytes in skin lesions has been described in psoriasis [29]. Here we report that this phenomenon is also present in APT-AE, AE and SE. Indeed, IL-18 has been shown to play a harmful effect to enhance inflammation in several autoimmune and inflammatory diseases [30]. Since B-cells are known to lack the expression of the IL-18 receptor, the effect of IL-18 on B-cell related activation has not been well studied. We suggest that there might be a direct or indirect interplay between IL-18 and BAFF/APRIL in AE, since the mRNA level of BAFF/APRIL correlates with that of IL-18 in lesional skin of AE and APT-AE. A connection between these factors is supported by a mouse model where daily i.p. injections of IL-18 for 10 days induce production of both IgE [31], [32] and BAFF (our own unpublished observation). Furthermore, we demonstrated that although IL-18 itself had no direct effect on the regulation of BAFF and APRIL production in cultured keratinocytes, IFN-γ dramatically increased the BAFF mRNA level in these cells. Since IL-18 is known to be a powerful stimulator of IFN-γ production [33], we therefore suggest that IL-18 could be produced locally by inflammatory cells and indirectly regulate the production of BAFF and APRIL in keratinocytes through IFN-γ.

Elevated serum levels of TWEAK have been found in patients with chronic inflammatory diseases and been suggested to be playing either a protective or harmful role [14]. For example, in systemic sclerosis where TWEAK is associated with a low frequency of pulmonary fibrosis [34] and in rheumatoid arthritis where the levels of TWEAK reflect disease activity [35]. In our patient cohort, we found no difference in the serum level of TWEAK in patients with AE or SE compared with HCs. This is in agreement with a recent study, where the serum level of TWEAK was reported to be similar among patients with AE or psoriasis and HCs [15]. Thus, unlike other chronic inflammatory diseases, increased level of TWEAK is not a candidate for a systemic role in the pathogenesis of AE and SE. Zimmermann *et al* also found that the TWEAK mRNA expression in healthy keratinocytes was not influenced by various eczema related stimuli [15] in agreement with our results. However, although the authors found similar mRNA expression of TWEAK between lesional and non-lesional skin from patients with AE, they found that the protein expression of TWEAK was only present in the epidermis of lesional, but absent in non-lesional skin by means of immunofluorescence [15]. In the biopsy specimens used in our study we could detect TWEAK expression not only in lesional AE skin, but also in healthy skin by immunofluorescence and we also found altered mRNA levels of TWEAK and Fn14 in AE lesions compared with HC. These differences between our study and the study by Zimmermann *et al* [15] could besides different immuno-staining protocols and source of anti-TWEAK and anti-Fn14 mAb possibly be due to the source of healthy skin; they used non-lesional skin from the buttock area of patients with AE or psoriasis, whereas we used skin biopsy specimens from healthy individuals. Furthermore, the source of the primary healthy keratinocytes was from adults in their study [15] and from young individuals in ours, which might lead to the different observations on the Fn14 expression in cultured keratinocytes by immunofluorescence. Another study also reported the protein expression of Fn14 on primary keratinocytes by FACS analysis [16], but the culture system in this study is different from what we used in this study; the extra supplements in their culture media could contribute to the induction of Fn14 expression in keratinocytes *ex vivo*. Further studies are needed to verify these possibilities.

It is known that the expression of Fn14 is inducible by tissues injury and that Fn14 signalling can occur in a TWEAK-dependent or -independent manner, contributing to tissue repair or a pathological effect depending on different disease or injury setting [14]. Indeed, we found that Fn14 was expressed at a low level in healthy skin and its expression level was significantly induced in the skin of AE and APT-AE. Importantly, we report here that in AE lesions the mRNA level of Fn14 correlates inversely with that of TWEAK, suggesting a compensatory mechanism that regulates TWEAK and Fn14 expression in AE lesions, where TWEAK-independent Fn14 signalling might be more dominate. Meanwhile, our data suggest that TWEAK-independent Fn14 signalling is not at work in SE lesions, where the TWEAK-dependent signalling could be more important in this setting, since the expression levels of TWEAK and Fn14 positively correlate to each other. Thus, we suggest that the persistent activation of Fn14 signalling in skin lesions of AE and SE could result in harmful and pathological effects to perpetuate the diseases. Therefore blocking TWEAK-independent and -dependent Fn14 signalling could be a therapeutic strategy for AE and SE, respectively. Whereas we also show here that blocking Fn14 signalling might not be suitable for the acute phase of AE, since the expression level of Fn14 correlates inversely with the APT score, suggesting a protective role of Fn14 signalling in acute AE. This suggestion is also supported by the current hypothesis that TWEAK-Fn14 axis normally plays a beneficial role in tissue repair following acute injury [14].

In conclusion, our data suggests that elevated levels of BAFF, APRIL or TWEAK in the circulation are not a part of the pathogenesis of AE and SE. However, the expression of these cytokines is altered in the local inflammatory environment in lesional skin of AE and SE. Furthermore, the expression pattern in AE lesions can be distinguished from that of SE lesions, as well as from that in the acute phase of AE lesions. Therefore, we propose that BAFF, APRIL and TWEAK can be considered as potential targets for the development of different therapeutic strategies in these inflammatory skin disorders.

## Materials and Methods

### Subjects and Ethics

Adult patients with AE or SE and HCs were recruited in the Stockholm area (Table 2). The inclusion and exclusion criteria for the AE patients were as previously described [36]. AE disease severity was assessed by the objective SCORAD index [37]. The SE patients had no history of additional skin disease. The HC had no clinical symptoms or history of allergy or skin disease. All participants gave their written informed consent. The study was approved by the Swedish regional ethics committee.

### Atopy patch test and skin biopsy specimens

APT with an in house extract from *M. sympodialis* (ATCC strain 42132) [38] was performed on non-lesional skin of AE patients as previously described [36]. The APT reaction was evaluated 48 h later and scored from 0 to 5+ on a scale proposed by European Task Force on Atopic Dermatitis [39]. Four mm punch skin biopsy specimens were taken under local anaesthesia from positive APT reactions (APT-AE), lesional skin from AE or SE patients and normal looking skin from HC. The specimens were snap-frozen and stored at −80°C until analysis.

### Serology

The concentrations of BAFF (R&D Systems, Minneapolis, MN) and APRIL (Bender Medsystems, Vienna, Austria) in plasma and TWEAK (Bender MedSystems) in sera were measured by ELISA according to the manufacturer's instruction. Total IgE (reference range 1.6–122 kU/L), *Malassezia*-specific IgE (m227, mixture of *M. sympodialis*, *M. globosa* and *M. restricta*, positive≥0.35 kU/L) and specific IgE to any of 11 common aeroallergens (Phadiatop®, positive≥0.35 kU/L) were measured in plasma using ImmunoCAP™ (Phadia AB, Uppsala, Sweden).

### Keratinocyte culture

Human keratinocytes originally isolated from skin of healthy young boys in connection with a routine surgical operation on the foreskin [40] were cultured in Keratinocyte-SFM® containing 5 µg/L human recombinant epidermal growth factor (EGF 1–53) and 50 mg/L bovine pituitary extract (BPE) (GIBCO® Invitrogen Cell Culture, Auckland, New Zealand) and 1% penicillin at 37°C in humidified atmosphere plus 5% CO_2_. The third- or fourth-passage keratinocytes were added to sterile microscope slides (Novakemi AB, Stockholm, Sweden) and incubated overnight to let the cells attach on the slides. The slides were then washed with PBS, air-dried and stored at −80°C until analysis. In addition, the keratinocytes were cultured in duplicates in 24-well plates (Becton Dickinson, Franklin lakes, NJ) for 24 h in 700 µl culture medium alone or stimulated with 10 µg/ml extract from *M. sympodialis* (ATCC strain 42132) [38], 10 µg/ml LPS (strain O111:B4, Sigma, St Louise, MO), 10 µg/ml Lipoteichoic acid (LTA, from *Staphylococcus aureus*, Sigma), 10 ng/ml rhIFN-γ (Sigma), 10 ng/ml rhTNF-α (R&D Systems, Minneapolis, MN), 10 ng/ml rhIL-4 (Invitrogen, Camarillo, CA), 100 ng/ml rhIL-18 (MBL International Corporation, Woburn, MA), 100 ng/ml rhIL-27 and 100 ng/ml rhIL-31 (R&D Systems). Total RNA of the keratinocytes was then isolated for quantitative QT-PCR analysis (see below).

### Quantitative RT-PCR

Total RNA from cultured keratinocytes or snap-frozen skin biopsies randomly selected from the participants (Table S1) was isolated by the TRIzol method (Invitrogen, Carlbad, CA). Isolated RNA was reversely transcribed by iScript TM cDNA synthesis kit (Bio-Rad, Hercules, CA). The cDNA was quantified in duplicates by means of real-time PCR with iQTM SYBR Green Supermix in the iCycler iQTM Multicolor real-time detection system (Bio-Rad). The primer sets for genes of interest were designed by Primer-BLAST program at NCBI databases. For each primer set (Table S2), non-template controls and melting curve were performed to control the specificity of the amplification. Relative expression level of a certain gene to the housekeeping gene GAPDH was calculated with the Pfaffl-formula [41].

### Immunofluorescence

Six-µm cryosections of skin biopsies and keratinocytes attached to slides were fixed in acetone. After blocking with goat serum, sections were incubated with primary mAb, followed by several washes in PBS and incubation with fluorescently-labelled secondary Abs. The following Abs were used: anti-BAFF (Buffy-2, rat IgM) and anti-APRIL (Aprily-8, mouse IgG_1_) (Axxora, Lausen, Switzerland); anti-TWEAK (CARL-1, mouse IgG_3_), anti-BAFFR (11c1, mouse IgG_1_), biotin conjugated anti-TACI (1A1, rat IgG_2a_) and anti-Fn14 (ITEM-1, mouse IgG_1_) (Biolegend, San Diego, CA); anti-IL-18 (25-2G, mouse IgG_1_; Medical & Biological Laboratories, Naka-ku Nagoya, Japan); Alexa-488 or 546-conjugated F(ab')_2_ of goat anti-mouse IgG and Alexa-488 or 647-conjugated goat anti-rat IgG (Invitrogen, Eugene, OR). In double-stainings, FITC or PE-conjugated anti-CD3 (SK7, mouse IgG_1_; BD biosciences, San Jose, CA) or Alexa-488-conjugated anti-CD68 (KP1, mouse IgG_1_; Santa Cruz Biotechnology, Heidelberg, Germany) were used. Stainings with isotype controls were performed to confirm the specificity of the immunostainings. Images were acquired using a confocal laser scanning microscope (TCS SP2; Leica Microsystems, Mannheim, Germany).

### Statistical analysis

Mann-Whitney test was used to test the difference between two groups. Correlation was assessed by Spearman's rank correlation. All calculations were performed by GraphPad Prism 5 software (GraphPad Software Inc., La Jolla, CA). p<0.05 was considered significant.

## Supporting Information

Table S1 Characterization of the patients with AE or SE and HC included in analysis of skin specimens.
(DOC)

Table S2 Genes of interest and primer sequences.
(DOC)
