# Supplementary material for: The Expression of BAFF, APRIL and TWEAK Is Altered in Eczema Skin but Not in the Circulation of Atopic and Seborrheic Eczema Patients
Source: PLoS One. 2011 Jul 13;6(7):e22202. doi: 10.1371/journal.pone.0022202 (PMC3135616; doi:10.1371/journal.pone.0022202)
Supplement: Table S1 — Characterization of the patients with AE or SE and HC included in analysis of skin specimens. (DOC) [file pone.0022202.s001.doc]

**Table S1:** Characterization of the patients with AE or SE and HC included in analysis of skin specimens

| Participant | Age  (years) | Gender | Plasma total IgE (kU/L) | Plasma *Malassezia*- IgE (kU/L) | Phadiatop® | Asthma and/or rhinitis | SCORAD | Score APT reaction |
| --- | --- | --- | --- | --- | --- | --- | --- | --- |
| AE 1 | 18 | female | 4500 | 100 | positive | yes | 59 | 3+ |
| AE 2^*^ | 29 | male | 1600 | 21 | positive | yes | 65 | 4+ |
| AE 3 | 44 | female | 72 | 2 | positive | yes | 35 | 4+ |
| AE 4 | 37 | male | 2900 | 27 | positive | yes | 50 | 3+ |
| AE 5 | 46 | male | 61 | <0.35 | positive | yes | 27 | 4+ |
| AE 6^*^ | 57 | male | 330 | 6.3 | positive | yes | 34 | 3+ |
| AE 7 | 44 | female | 10600 | 13 | positive | yes | 58 | 0 |
| AE 8 | 42 | male | 9800 | 38 | positive | yes | 64 | 0 |
| AE 9^#^ | 19 | female | 17 | <0.35 | negative | no | 37 | 0 |
| AE 10 | 57 | female | 2600 | 35 | positive | yes | 31 | 0 |
| AE 11 | 49 | male | 2700 | 21 | positive | yes | 42 | 5+ |
| AE 12 | 23 | female | 4.5 | <0.35 | negative | yes | 51 | 0 |
| AE 13^#^ | 20 | female | 2300 | 100 | positive | no | 34 | 1+ |
| AE 14^#^ | 43 | male | 1700 | 37 | positive | yes | 55 | 3+ |
| AE 15 | 30 | male | 84 | <0.35 | positive | yes | 20 | 0 |
| SE 1^*^ | 32 | male | 9.5 | <0.35 | negative | no |  |  |
| SE 2 | 44 | male | 17 | <0.35 | negative | no |  |  |
| SE 3 | 27 | male | 6 | <0.35 | negative | no |  |  |
| SE 4^*^ | 48 | female | 17 | <0.35 | negative | no |  |  |
| SE 5 | 48 | male | 12 | <0.35 | negative | no |  |  |
| SE 6 | 45 | male | 18 | <0.35 | negative | no |  |  |
| HC 1 | 61 | female | 29 | <0.35 | negative | no |  |  |
| HC 2 | 50 | male | 24 | <0.35 | negative | no |  |  |
| HC 3^#^ | 20 | male | 17 | <0.35 | negative | no |  |  |
| HC 4^#^ | 41 | female | 7.6 | <0.35 | negative | no |  |  |
| HC 5 | 41 | male | 77 | <0.35 | negative | no |  |  |
| HC 6 | 23 | male | 73 | <0.35 | negative | no |  |  |
| HC 7^#^ | 24 | male | 31 | <0.35 | negative | no |  |  |
| HC 8 | 23 | female | 11 | <0.35 | negative | no |  |  |
| HC 9 | 25 | female | 110 | <0.35 | positive | no |  |  |

Skin specimens from all participants were analyzed by quantitative RT-PCR (AE 1-6: APT reactions, AE 7-15: lesional skin). *, The same skin biopsy was analyzed by both quantitative RT-PCR and immunofluorescence; ^#^, Two separate skin biopsies were analyzed by quantitative RT-PCR and immunofluorescence, respectively.
