# Supplementary material for: The Expression of BAFF, APRIL and TWEAK Is Altered in Eczema Skin but Not in the Circulation of Atopic and Seborrheic Eczema Patients
Source: PLoS One. 2011 Jul 13;6(7):e22202. doi: 10.1371/journal.pone.0022202 (PMC3135616; doi:10.1371/journal.pone.0022202)
Supplement: Table S2 — Genes of interest and primer sequences. (DOC) [file pone.0022202.s002.doc]

**Table S2:** Genes of interest and primer sequences

| Gene | Forward primer sequence (5'- >3') | Reverse primer sequence (5'- >3') |
| --- | --- | --- |
| APRIL | CAGTTGCCCTCTGGTTGAGT | GAGGCTCTGCAGCTCTGTTT |
| BAFF | GGAGAAGGCAACTCCAGTCA | GCAATCAGTTGCAAGCAGTC |
| BAFFR | ATCTCTGATGCCACAGCTCC | GTGGTCACCAGTTCAGTGGA |
| CD19 | TACTATGGCACTGGCTGCTG | AAGGGAACACAGGCAGAAGA |
| Fn14 | TCTGGCTTTTTGGTCTGGAG | GGCACATTGTCACTGGATCA |
| GAPDH | AGGGCTGCTTTTAACTCTGGTAAA | CATATTGGAACATGTAAACCATGTAGTTG |
| IL-18 | CCAAGGAAATCGGCCTCTAT | GCCATACCTCTAGGCTGGCT |
| TACI | CATCTGTGGACAGCACCCTA | CTCCTGAGCTCTGGTGGAAG |
| TWEAK | TCAGGTGCACTTTGATGAGG | CTGAGAATTCCTCCAGGCAG |
| TWE-PRIL | GGAGGAAGCCAGAATCAACA | ATCTCTCCCCATTCTCCCAG |
